# miR-155 Is a Positive Regulator of FcεRI-Induced Cyclooxygenase-2 Expression and Cytokine Production in Mast Cells

**DOI:** 10.3389/falgy.2022.835776

**Published:** 2022-04-18

**Authors:** Zahraa Mohammed, Cody McHale, Jason L. Kubinak, Stuart Dryer, Gregorio Gomez

**Affiliations:** ^1^Department of Pathology, Microbiology and Immunology, University of South Carolina School of Medicine, Columbia, SC, United States; ^2^Molecular Targeted Therapeutics Laboratory, Levine Cancer Institute, Charlotte, NC, United States; ^3^Department of Biology and Biochemistry, College of Natural Sciences and Mathematics, University of Houston, Houston, TX, United States; ^4^Department of Biomedical Sciences, College of Medicine, University of Houston, Houston, TX, United States

**Keywords:** allergy, *COX-2*, Akt, FcεRI, miR-155, mast cells

## Abstract

MicroRNA-155 (miR-155) has been implicated in IgE-dependent allergic disease including asthma and atopic dermatitis. A few roles for miR-155 have been described in mast cells and some specifically related to IgE receptor signaling, but it is not completely understood. Here, we demonstrate by miRNA seq profiling and quantitative RT-PCR that miR-155 expression is significantly increased in human skin-derived mast cells (SMCs) and mouse bone marrow-derived mast cells (BMMCs) following FcεRI crosslinking with antigen. We demonstrate that FcεRI-induced expression of *cyclooxygenase-2* (*COX-2*) was significantly inhibited in miR-155 knockout (KO) BMMCs whereas *arachidonate-5-lipoxygenase* (*ALOX-5*) expression and leukotriene C4 (LTC_4_) biosynthesis, and degranulation were unaffected. FcεRI-induced cytokine production (TNF, IL-6, and IL-13) from miR-155 KO BMMCs was also significantly diminished. Correspondingly, Akt phosphorylation, but not protein expression, was inhibited in the absence of miR-155 whereas p38 and p42/44 were unaffected. Interesting, lipopolysaccharide (LPS)-induced cytokine production was increased in miR-155 KO BMMCs. Together, these data demonstrate that miR-155 specifically targets the FcεRI-induced prostaglandin and cytokine pathways, but not the leukotriene or degranulation pathways, in mast cells. The data further suggest that miR-155 acts indirectly by targeting a repressor of *COX-2* expression and a phosphatase that normally blocks Akt phosphorylation. Overall, this study reveals the role of miR-155 as a positive regulator of mast cell function.

## Introduction

Mast cells are widely recognized as the main effector cell type of allergic reactions including asthma (1–5). The effector function of mast cells is the result of the action of inflammatory mediators that are released upon activation. Mast cells can be activated in different ways, but allergic reactions including bronchoconstriction are associated with crosslinking of FcεRI, the high affinity receptor for IgE, with allergen. Upon activation, mast cells immediately release pre-formed mediators like histamine, serine neutral proteases, and proteoglycans from cytoplasmic granules where they are normally stored (6). The mediators that are released during “degranulation” are responsible for immediate hypersensitivity reactions including anaphylactic responses to food and insect venom. Within the same timeframe as degranulation (minutes), mast cells also biosynthesize and secrete inflammatory lipids including Prostaglandin D2 (PGD_2_) and cysteinyl leukotrienes (LTC_4_, LTD_4_, and LTE_4_), both of which play a major role in asthma pathogenesis (7–9). In fact, LTC_4_ and LTD_4_ are considered to be the most potent inducers of bronchoconstriction in asthma (10, 11). Crosslinking of FcεRI on mast cells also leads to the activation of transcription factors that induce the *de novo* production of many different inflammatory cytokines that contribute to delayed-type allergic inflammation (12). Thus, the pathophysiological effects of mast cells are dependent on the vast array of inflammatory mediators that they produce, release, and secrete upon activation.

MicroRNAs (miRNAs) are short (19–25 nucleotides) non-coding RNAs that are involved in post-transcriptional silencing of gene expression through the degradation of mRNAs (13). miRNAs act by associating *via* their 5′ end with an RNA-induced silencing complex (RISC) containing the protein Argonaute and other accessory proteins to target mRNAs (14). When the miRNA-RISC-Argonaute complex binds to a target mRNA, RISC acts to reduce the rate of translation and accelerate the shortening of the poly-A tail leading to faster degradation of the mRNA (13, 15, 16). It is estimated that over 30% of human genes are targeted by miRNAs (16, 17). Thus, miRNAs have the potential to significantly regulate susceptibility to disease. The notion that miRNAs can regulate atopy is supported by the observation that knocking down Dicer, which processes pre-miRNA into mature miRNA, not only blocked miRNA expression but also prevented FcεRI-mediated mast cell degranulation (18, 19). Studies have shown that miRNAs can regulate mast cells (20), and several have been implicated in susceptibility to allergy and asthma (21).

In our miRNA profiling studies in human skin mast cells, we identified 10 miRNAs that were significantly upregulated and 11 that were significantly downregulated following FcεRI crosslinking with multivalent allergen. Among those that were upregulated was miR-155, which has been implicated in asthma and other allergic diseases in humans (21). The finding that miR-155 expression was increased in human SMCs following FcεRI crosslinking was intriguing because miR-155 is known to be involved in the immune response (22) and has been implicated in allergic disease in humans including atopic dermatitis (23), allergic rhinitis (24), and asthma (25). Indeed, increased levels of miR-155 were observed in mast cells from skin lesions of patients with atopic dermatitis (23), in nasal biopsies of patients with allergic rhinitis (24), and in asthmatic airways and plasma (26, 27). In addition, allergen-challenged miR-155 knockout mice exhibited attenuated allergen-induced airway inflammation compared to wild-type (WT) mice (25). These observations suggest that miR-155 expression in mast cells could potentially control susceptibility to asthma and other allergic diseases; thus, suggesting that miR-155 plays a critical role in mast cell function. Interestingly, however, it was reported that miR-155 expression was not affected by IgE receptor crosslinking on mouse BMMCs (28). This prompted us to investigate the FcεRI-induced expression of miR-155 and its role in mast cell mediator release. Some studies have provided insight. For example, it was shown that miR-155 induced by IL-10 enhanced IgE-dependent cytokine production by targeting suppressor of cytokine signaling 1 (SOCS1) (29), and miR-155 was also shown to control mast cell activation by targeting the PI3Kγ pathway (28). In addition, miR-155 inhibited IL-33-induced cytokine production in mast cells (30). However, the exact role of miR-155 in mast cells is not completely understood. Therefore, to further investigate, the present study utilized BMMCs from miR-155 KO mice to determine the role of miR-155 on FcεRI mediator release from mast cells.

## Materials and Methods

### Murine Bone Marrow-Derived Mast Cells

Murine mast cells were derived from bone marrow of sex and age-matched (8–12 weeks old) miR-155^−/−^ and wild type C57BL/6 mice that were generously provided by Dr. Daping Fan and Dr. Angela Murphy (University of South Carolina School of Medicine). The mice were housed and used in accordance with animal use protocols approved by the Institutional Animal Care and Use Committee (IACUC) of the University of South Carolina. Original breeder pairs were purchased from The Jackson Laboratory and bred in-house. The bone marrow was flushed from femurs and tibias with RPMI 1640 media and passed through a 40 μm filter before collecting the cells in a 50 ml conical tube. Total bone marrow cells were washed twice with RPMI 1640 media and resuspended at 10^6^ cells/ml in complete RPMI 1640 media supplemented with 10% fetal calf serum (FCS) and 10 ng/ml each of murine recombinant stem cell factor (SCF) and interleukin-3 (IL-3). The single cell suspensions were transferred to a tissue culture flask and maintained under standard culture conditions (37°C, 5% CO_2_) with weekly media changes. The mast cells were monitored weekly for monochromatic staining with toluidine blue and were used for experiments when >95% of the cells were FcεRI^+^. For IgE-dependent activation studies, BMMCs were first sensitized to the antigen 2,4-Dinitrophenyl (DNP) by incubating overnight with anti-DNP IgE (0.1 μg/10^6^ cells), generously provided by Dr. Daniel Conrad (Virginia Commonwealth University), in complete culture media. After the sensitization period, the BMMCs were washed 3 × with complete culture media or Tyrode's buffer (135 mM NaCl, 1 mM MgCl_2_, 20 mM Hepes, 5 mM KCl, 1.8 mM CaCl_2_, 5.6 mM glucose; pH 7.4) to remove unbound IgE, resuspended, and then activated at 37°C with DNP conjugated to human serum albumin (DNP-HSA) at the indicated concentrations and time points.

### Human Skin Mast Cells

SMCs were isolated and purified from fresh surgical specimens of human skin tissue, which were purchased from the Cooperative Human Tissue Network (CHTN) of the National Cancer Institute as approved by the human studies Institutional Review Board (IRB) of University of South Carolina. The study was deemed exempt from Protection of Human Subjects Research regulations and does not qualify as human subjects research. Subjects from whom tissues are obtained by the CHTN are consented by that organization. Subject identifiers are not provided to the investigators. Skin tissues were processed and mast cell isolated as described in our recent studies (31, 32). Basically, the skin tissue was mechanically disrupted with surgical scissors and then digested 3 × 1 h at 37°C with collagenase type II, hyaluronidase from bovine testes, and DNase I in Hanks Balanced Salt Solution (HBSS) wash buffer (1X HBSS, 0.04% NaHCO3, 1% fetal bovine serum, 1% HEPES, and 0.1% CaCl2) containing amphotericin B and Antibiotic/Antimycotic solution. After each digestion period, the samples were filtered through 40 μm nylon cell strainers, washed, and re-suspended in wash buffer. After the final digestion, the cells were separated on a Percoll gradient by density centrifugation. The cells at the interface of buffer and Percoll layers were collected and washed with culture media. Purified single-cell suspensions of SMCs were cultured in X-VIVO 15™ media containing SCF (100 ng/ml) with weekly media changes. Purity was assessed by metachromatic staining with acidic toluidine blue, which stains the mast cell granules purple, and by immunofluorescence for FcεRI expression with phycoerythrin (PE)-labeled anti-human FcεRI antibody (clone AER-37 (CRA) and IgG2b_k_ isotype control. SMCs were used in experiments when >95% were FcεRI^+^ (BioLegend, San Diego CA, USA). For IgE-dependent activation, SMCs (10^6^ cells/ml) were sensitized to the antigen 4-hydroxy-3-nitrophenylacetyl (NP) by incubating overnight with chimeric human anti-NP IgE (1 μg/ml) (clone JW8/1; Bio-Rad (formerly AbD Serotec), Hercules CA, USA) in X-VIVO 15™ + SCF (100 ng/ml) at 37°C. After the sensitization period, the SMCs were washed 3 × with X-VIVO 15™ to remove unbound IgE, resuspended at 10^6^ cells/ml in X-VIVO 15™ + SCF (100 ng/ml), and then activated at 37°C with NP conjugated to bovine serum albumin (NP-BSA) (Biosearch Technologies, Novato CA, USA) at the indicated concentrations and time-points.

### miRNA Profiling

miRNA profiling services were performed by LC Sciences (lcsciences.com, Houston TX, USA) using their μParaflo^®^ microfluidic biochip technology and optimized probes in a microfluidics microarray platform. To prepare samples, human skin mast cells were sensitized to the antigen NP by incubating with NP-specific IgE and then activated, or not, with the multivalent antigen NP-BSA (1 ng/ml) for 3 h, as described above. After the activation period, SMCs were separated from supernatant by centrifugation and RNA was extracted using basic miRNeasy kits (Qiagen, Germantown MD, USA). The RNA samples were snap frozen in liquid nitrogen and shipped on dry ice for analysis. The MicroRNA Expression Profiling services included: sample quality control (QC), sample preparation, microRNA detection, array scan and data extraction, and full data analysis.

### Degranulation, Cytokine, and LTC_4_ Release Assays

FcεRI-induced degranulation was determined by standard β-hexosaminidase release assay and LTC4 release by commercial enzyme immunoassay. IgE-sensitized BMMCs (10^6^ cells/ml) were activated with DNP-HSA at the indicated concentrations for 30 min in Tyrode'sbuffer containing 0.05% BSA. After the activation period, BMMCs and supernatant were separated by centrifugation, and BMMCs were lysed with 1% Trixon X-100. For degranulation, 10 μl of supernatant or lysate was mixed with 10 μl of 1 mM p-nitrophenyl N-acetyl-β-D-glucosaminide (PNAG; Sigma-Aldrich, St. Louis MO, USA) in a 96 well-plate and incubated for 1 h at 37°C. The reaction was terminated, and color change induced with 200 μl/well of 0.1 M Na_2_CO_3_/NaHCO_3_ buffer, and absorbance was read at 450 nm. Percent β-hexosaminidase release was calculated from the absorbance values according to the formula: % β-hexosaminidase release = [(supernatant)/(supernatant + lysate)] × 100. LTC_4_ in the supernatant was measured with commercial enzyme immunoassay (Cayman Chemical; Ann Arbor MI, USA) according to the manufacturer's instructions. For cytokine determination, BMMCs (10^6^ cells/ml) were activated with IgE/Ag as described above or with LPS at the indicated concentrations for 24 h in complete RPMI 1640 media supplemented with SCF and IL-3. Tumor Necrosis Factor (TNF), IL-6, and IL-13 in the cell-free media were measured with commercial Mouse DuoSet enzyme linked immunosorbent assay (ELISA) kits (R&D Systems; Minneapolis MN, USA). Absorbance measurements were taken on a BioTek Synergy HT microplate reader, and cytokine concentrations were determined using Gen5 Data Analysis Software.

### Gene Expression Analysis

Gene expression was determined by quantitative real-time PCR using *SNORD96A* as the reference gene. IgE-sensitized BMMCs were activated or not with DNP-HSA at the indicated concentrations and time-points, and RNA was extracted with miRNeasy kits. For miR-155 analysis, cDNA was synthesized with miScript II RT with HiFlex buffer, and PCR was carried out with miScript SYBR Green and miScript Primer Assays for human and mouse miR-155-5p and *SNORD96A* as the control gene. PCR was carried out with 2 ng of cDNA per reaction in a hot start protocol: [95°C × 15 min, (94°C × 15 s, 55°C × 30 s, and 70°C × 30 s) × 35 cycles]. All reagents used for miRNA gene expression analysis were purchased from Qiagen and used according to the manufacturer's instructions. For *COX-2* and *ALOX5* analysis, cDNA was synthesized with the iScript cDNA Synthesis kit, and PCR was performed using iQ SYBR^®^ Green Supermix (Bio-Rad). PCR was carried out with 200 ng of cDNA per reaction in a hot-start protocol: [95°C × 5 min, (95°C × 30 s, 55°C × 30 s, and 72°C × 30 s) × 35 cycles, 95°C × 1 min, and 55°C × 1 min]. Pre-designed and validated oligonucleotide primers (Sigma-Aldrich) used: *COX-2* (F: 5′-ACTGCTCAACACCGGAATTT-3′, R: 5′-CAAGGGAGTCGGGCAATCAT-3′), *ALOX5* (F: 5′-CAGGAAGGGAACATTTTCATC-3′, R: 5′-AGGAAGATTGGGTTACTCTC-3′), and β*2 microglobulin* (B2M) (F: 5′-TGGGTTTCATCCATCCGACA-3′, R: 5′-CTGCTTACATGTCTCGATCCC-3′). Analysis was performed on a CFX Connect Real Time PCR Detection System (Bio-Rad). Fold change in expression was determined by the 2^ΔΔCt^ method.

### miR-155 Genotyping

miR-155 genotyping was performed using a modified version of a protocol provided by Jackson Laboratories (jax.org). Genomic DNA was extracted from WT and miR-155 KO BMMCs by incubating with DirectPCR Lysis Reagent (cell) + Proteinase K solution (Viagen Biotech; Los Angeles CA, USA) overnight in a 55°C water bath followed by a 1 h incubation at 85°C. The DNA was precipitated with ethanol + sodium acetate (NaOAc), resuspended in water, and 50 ng per reaction was amplified with iTaq Universal SYBR Green Supermix (Bio-Rad) in a reaction mix containing wild type (5′-AATCATTCCTGAGGG CTACC-3′) or mutant (5′-GCCTGAAGAACGAGATCAGC-3′) forward primer and a common primer (5′-GGAAACGTGGGTCTCCTTAC-3′) with the protocol 94°C × 5 min, (94°C × 5 min, 61.8°C × 1 min, 72°C × 30 s) × 36 cycles, 72°C × 3 min. For visualization, the PCR products were loaded onto a 1.5% Tris-Borate-EDTA (TBE) gel containing ethidium bromide and electrophoresed. The expected band sizes were 165 bp for *miR-155*^+/+^ and 226 bp for *miR-155*^−/−^.

### Flow Cytometry

BMMCs (10^6^/ml) were washed and resuspended in FACS buffer [1% BSA, 0.04% sodium azide (NaN_3_) in PBS] on ice. FcγRs were blocked with rat anti-mouse CD16/32 (Clone S17011E) (1 μg/10^6^ cells) for 20 min on ice. The cells were stained with fluorescein isothocyanate (FITC)-labeled anti-mouse FcεRIα mAb (clone MAR-1) or IgG isotype control (clone HTK888) (BioLegend) (1 μg/10^6^ cells) for 20 min on ice. The cells were washed twice in FACS buffer and fixed with 2% paraformaldehyde. Data was collected using a FACSAria II cell sorter, and analyzed with FlowJo v10 software (FlowJo, LLC; Ashland OR, USA).

### Immunoblotting

Whole cell lysates were prepared from BMMCs that were activated as indicated. BMMC activation was terminated immediately by the addition of ice-cold PBS. The BMMCs were pelleted by centrifugation and lysed (10^7^ cells/ml) with Tris-Glycine SDS Sample Buffer (Life Technologies, Carlsbad CA, USA) containing 1% β-mercaptoethanol and 1 mM Na_3_VO_4_. Equivalent volumes were loaded onto 10–12% Tris-Glycine polyacrylamide gels and separated by SDS-PAGE. The separated proteins were then transferred onto nitrocellulose membranes with Towbin's Transfer Buffer (25 μM Tris, 192 mM Glycine, and 20% Methanol) using a semi-dry transfer apparatus (Bio-Rad). After transfer, the membranes were blocked for 1 h at room temperature with Odyssey Blocking Buffer (LI-COR Biosciences; Lincoln, NE, USA). Two-color staining was performed by incubating the blots overnight at 4°C with the following combination of primary antibodies (Cell Signaling Technology; Danvers, MA, USA): rabbit polyclonal anti-p38 MAPK + mouse monoclonal anti-phospho-p38 MAPK (Thr180/Tyr182) (28B10), rabbit polyclonal anti-p44/42 (Erk 1/2) + mouse monoclonal anti-phospho-p42/44 (Erk1/2) (Thr202/Tyr204) (E10), rabbit polyclonal anti-Akt + mouse monoclonal anti-phospho-Akt (Thr308) (L32A4), or rabbit monoclonal anti-ALOX5 (C49G1) + mouse monoclonal anti-β-actin (8H10D10). After the incubation period, the blots were washed and incubated for 1 h at room temperature with secondary antibodies goat anti-rabbit IRDye 680RD and goat anti-mouse 800CW (LI-COR Biosciences). The blots were then washed and scanned on an Odyssey^®^ CLx Infrared Imaging System and analyzed with Image Studio Software version 3.1.4 (LI-COR Biosciences).

### Statistical Analysis

Statistical significance was determined by Student's *t*-test as indicated in the figure legends. Statistical analysis was performed using GraphPad Prism version 6.0 for Mac OS X, GraphPad Software, La Jolla, CA, USA.

## Results

### FcεRI Crosslinking Modulates miRNA Expression in Human SMCs

**Figure 1:**
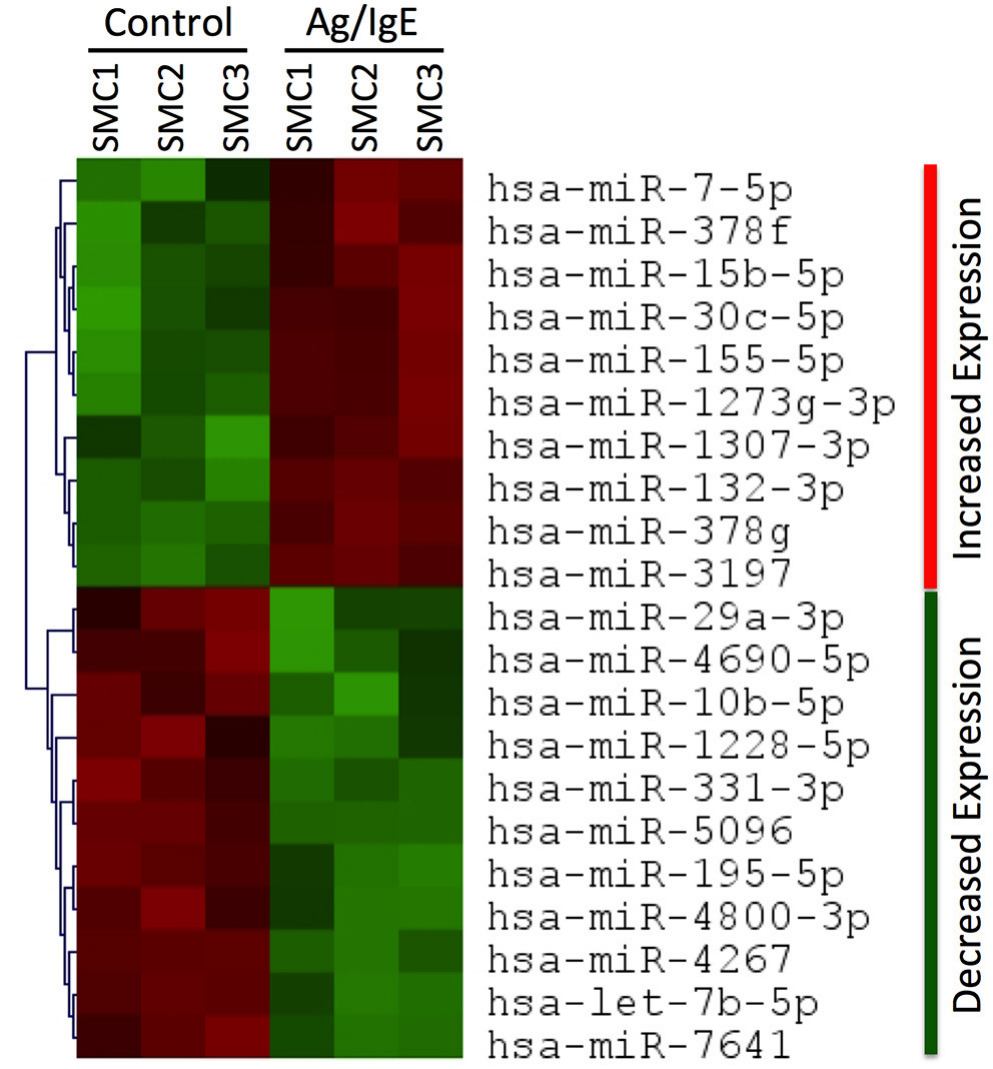
miRNA profiling in human SMCs. Heat map showing significant (*p* < 0.01) changes in miRNA expression in human SMCs following FcεRI crosslinking. RNA was extracted from SMCs that were sensitized with chimeric human anti-NP IgE (1 μg/ml) and activated for 3 h at 37°C with the multivalent antigen NP-HSA (1 ng/ml). The heat map identifies 10 miRNAs that were significantly (*p* < 0.01) upregulated (red) and 11 that were significantly downregulated (green) in Ag/IgE-activated SMCs (*n* = 3 different cultures from different donors). All data can be found in the Supplementary Material.

To determine if FcεRI crosslinking was sufficient to alter the expression profile of miRNAs in human tissue-derived mast cells, we performed miRNA seq analysis on human SMCs that were sensitized with anti-NP IgE and activated, or not, with a low dose of the multivalent antigen NP-BSA. We identified 10 miRNAs that were significantly (*p* < 0.01) upregulated and 11 that were significantly downregulated following FcεRI crosslinking (Figure 1). The up-regulated miRNAs were miR-7-5p, miR-378f, miR-15b-5p, miR-30c-5p, miR-155-5p, miR-1273g-3p, miR-1307-3p, miR-132-3p, miR-378g, and miR-3197. The down-regulated miRNAs were miR-29a-3p, miR-4690-5p, miR-10b-5p, miR-1228-5p, miR-331-3p, miR-5096, miR-195-5p, miR-4800-3p, miR-4267, let-7b-5p, and miR-7641.

### FcεRI Crosslinking Upregulates miR-155 Expression in Human and Mouse Mast Cells

**Figure 2:**
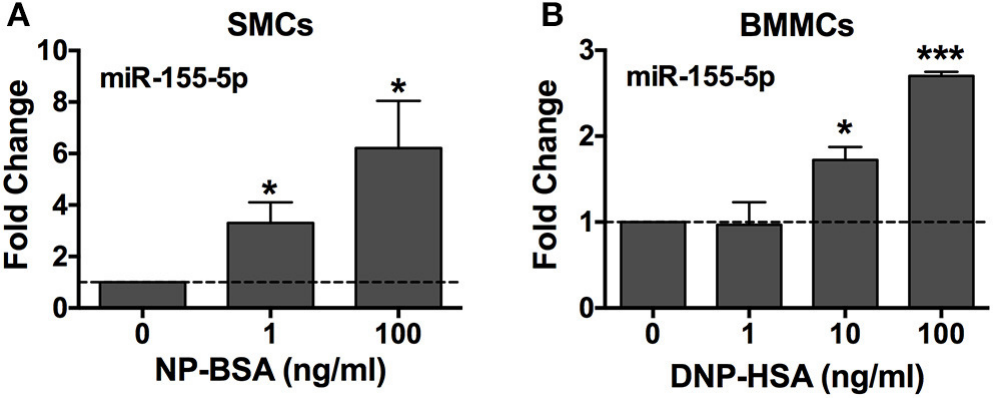
FcεRI crosslinking induces miR-155-5p expression in human and mouse mast cells. Human SMCs isolated from tissues of different donors (**A**, *n* = 3) and mouse BMMCs from male C57BL6 mice (**B**, *n* = 3) were sensitized with anti-NP IgE or anti-DNP IgE, respectively, and activated for 3 h at 37°C with 1–100 ng/ml of the multivalent antigen NP-BSA or DNP-HSA. Changes in miR-155-5p expression were determined by quantitative RT-PCR using the 2^ΔΔCq^ method with *SNORD96A* as the internal reference control. The bar graphs represent mean ± SEM of values obtained from independent experiments with three different BMMC or SMC cultures from different mice or human donors. Significance was determined with Student's *t*-test. *p < 0.05; ***p < 0.001.

To confirm the miRNA seq data, we analyzed miR-155 expression by quantitative real-time PCR in human SMCs and mouse BMMCs. Mast cells were sensitized with anti-NP IgE (human) or anti-DNP IgE (mouse) and activated, or not, with the multivalent antigen NP-BSA (human) or DNP-HSA (mouse) for 3 h. miR-155 expression was shown to be significantly upregulated in a dose-dependent manner in both human and mouse mast cells after crosslinking FcεRI with multivalent antigen (Figure 2). These data confirm the miRNA profiler data for miR-155.

### miR-155 Deficiency Does Not Affect FcεRI Expression or Granule Content in Mast Cells

**Figure 3:**
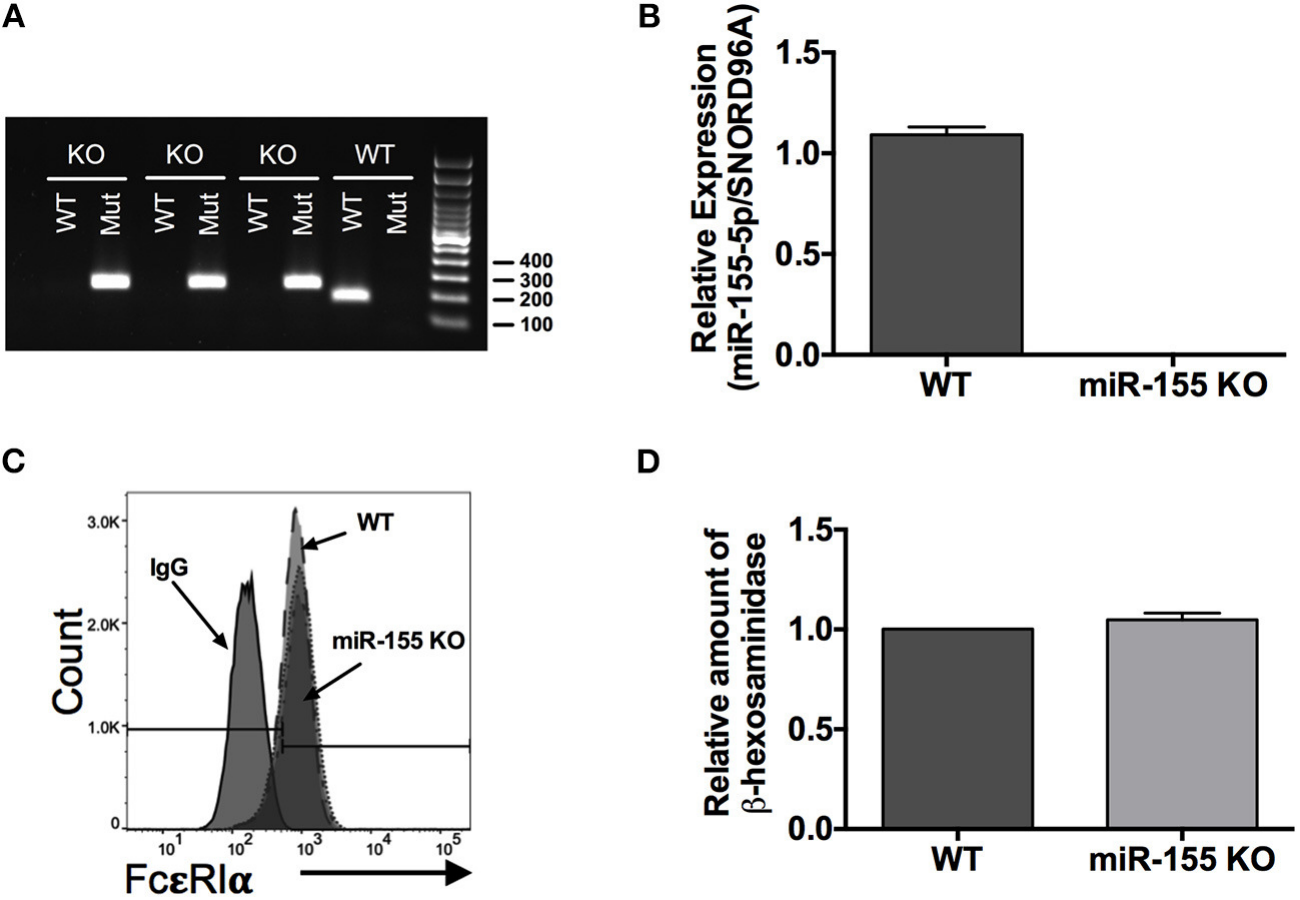
Genotyping and characterization of miR-155 knockout (KO) BMMCs. **(A)** Representative DNA gel of PCR products. Genotyping was routinely performed on all BMMC cultures to confirm miR-155 deficiency. **(B)** Quantitative RT-PCR analysis of miR-155-5p expression in wild-type and miR-155 KO BMMCs. **(C)** Representative flow cytometry histogram of surface FcεRI expression on wild-type and miR-155 KO BMMCs. **(D)** Bar graph showing relative amount of total β-hexosaminidase in miR-155 BMMCs compared to wild-type BMMCs. The bar graphs represent mean ± SEM of values obtained from independent experiments with three different BMMC cultures from different mice. Significance was determined with Student's *t*-test.

To investigate the role of miR-155 on IgE-dependent mediator release from mast cells we used BMMCs that were generated from wild-type and miR-155 knockout (KO) mice. Throughout the study and prior to experimentation, we confirmed the genotype of the BMMCs by PCR (Figure 3A) and quantitative RT-PCR (Figure 3B). We further demonstrated by flow cytometry that surface expression of FcεRI was the same on wild type and miR-155 KO BMMCs (Figure 3C). Mean fluorescence Intensity (MFI) values for WT and miR-155 KO BMMCs, respectively, was 991 and 906. In addition, the amount of total β-hexosaminidase was the same in BMMCs from both genotypes (Figure 3D). Thus, miR-155 deficiency did not affect FcεRI expression or granule content in mast cells.

### miR-155 Deficient BMMCs Exhibit Diminished FcεRI-Induced COX-2 Expression but Normal IgE-Dependent Degranulation and Cysteinyl LTC_4_ Biosynthesis

**Figure 4:**
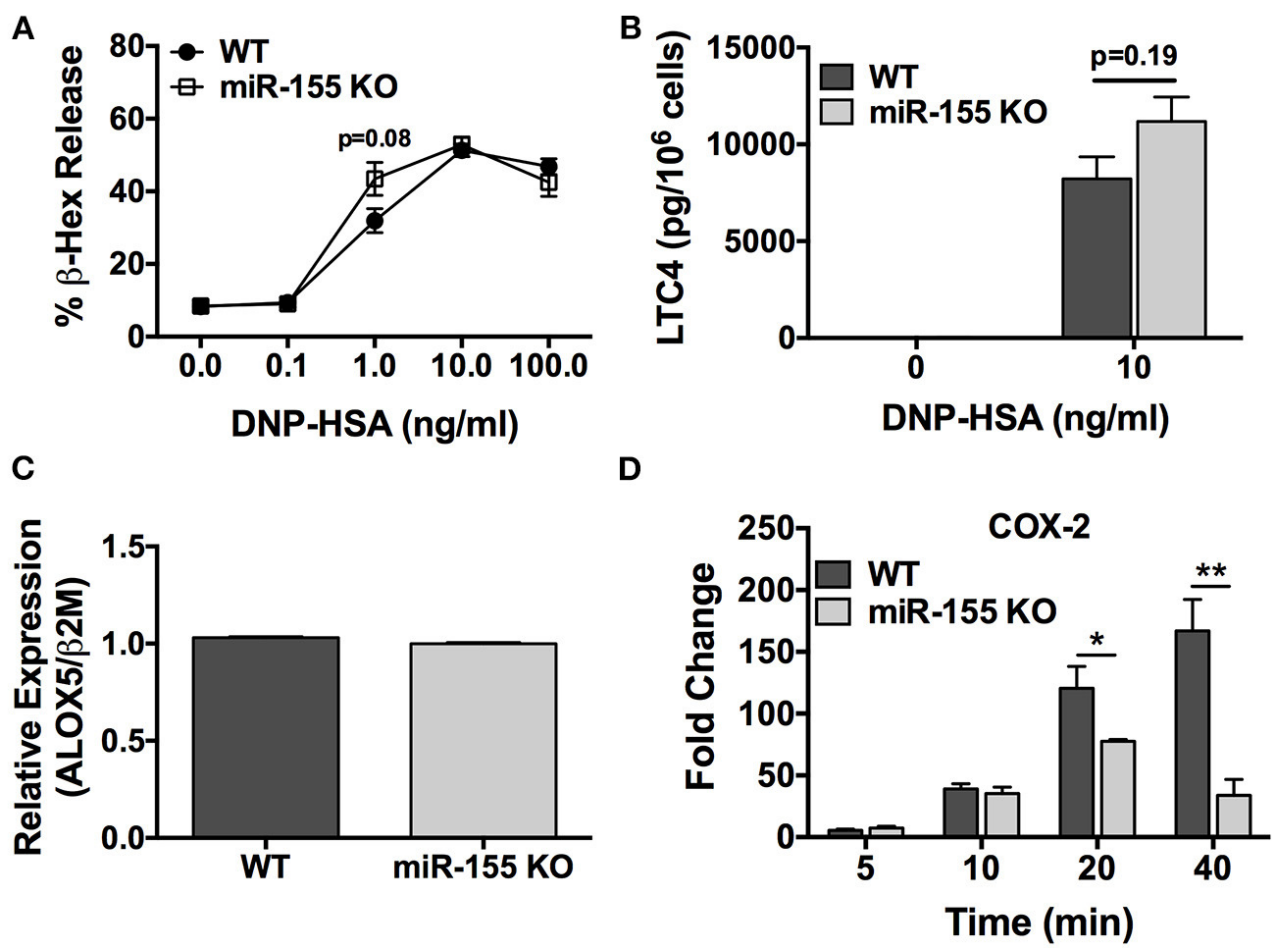
FcεRI-induced *COX-2* expression, but not degranulation, is impaired in miR-155 KO BMMCs. Wild-type and miR-155 KO BMMCs were sensitized with anti-DNP-IgE and challenged with multivalent antigen DNP-HSA. **(A)** Dose-dependent degranulation response of wild-type and miR-155 KO BMMCs after 30 min activation period. Degranulation was determined by β-hexosaminidase release. **(B)** Leukotriene C4 (LTC_4_) biosynthesis after 30 min activation period. **(C)** Expression of *ALOX5* in non-activated wild-type and miR-155 KO BMMCs. Analysis performed was quantitative RT-PCR. **(D)** Time-course for FcεRI-induced *COX-2* expression in wild-type and miR-155 KO BMMCs. Fold change in COX-2 expression was determined by quantitative real-time PCR. The figures represent mean ± SEM of values obtained from independent experiments with 3–6 different BMMC cultures of each genotype from different mice. Significance was determined with Student's *t*-test. *p < 0.05; **p < 0.01.

To determine the role of miR-155 on IgE-dependent degranulation, wild type and miR-155 KO BMMCs were sensitized with anti-DNP IgE and then challenged with DNP-HSA (0.1–100 ng/ml) for 30 min in dose-response experiments. Degranulation was determined by β-hexosaminidase release assay. As shown in Figure 4A, FcεRI-mediated activation induced a robust dose-dependent degranulation response from miR-155 KO BMMCs that was practically identical to that observed from WT BMMCs, thus, demonstrating that miR-155 does not affect the ability of mast cells to degranulate in response to FcεRI crosslinking.

Next, we determined the effect of miR-155 deficiency on FcεRI-induced biosynthesis of LTC_4_, one of the most important inducers of bronchoconstriction in asthma together with LTD_4_ (7–9). IgE-sensitized BMMCs were activated with DNP-HSA (10 ng/ml) for 30 min and secreted LTC_4_ in the supernatant was quantified. As shown in Figure 4B, LTC_4_ secretion from miR-155 deficient BMMCs was not significantly different than from WT mast cells. Accordingly, *ALOX5*, which encodes a key enzyme in the leukotriene pathway, was expressed at similar levels in miR-155 KO and WT BMMCs (Figure 4C) indicating that the leukotriene pathway is not targeted by miR-155 in mast cells.

We then sought to determine the effect of miR-155 deficiency on biosynthesis of PGD_2_, which is produced in significant amounts by mast cells (33, 34) and is involved in inflammation and asthma pathogenesis (7). Unfortunately, PGD_2_ could not be measured directly because to our knowledge the available commercial PGD_2_ ELISA kit utilizes an anti-mouse Ig and, thus, is not recommended for use with mouse samples due to the potential for interference. Therefore, in lieu of this, we quantified the FcεRI-induced expression of *COX-2*, a key inducible enzyme in the prostaglandin pathway that is directly involved in PGD_2_ biosynthesis. In time-course experiments, IgE-sensitized BMMCs were activated with DNP-HSA (10 ng/ml) and *COX-2* expression was determined at 5, 10, 20, and 40 min (Figure 4D). As shown, *COX-2* expression in WT cells increased in a time-dependent manner and was significantly greater than the induced expression in miR-155 KO BMMCs at 20 and 40 min following activation. Whereas, *COX-2* expression in WT cells continuously increased over time, the expression in miR-155 KO BMMCs peaked at 20 min and was diminished at 40 min. Thus, FcεRI-induced expression of *COX-2* was defective in miR-155 KO BMMCs.

Taken together, these data reveal that miR-155 does not control IgE-dependent degranulation or the leukotriene pathway. However, miR-155 specifically but indirectly targets the prostaglandin pathway in mast cells to positively regulate FcεRI-induced *COX-2* expression leading to PGD_2_ biosynthesis. Thus, the data suggest that the target of miR-155 is an inhibitor of *COX-2* expression.

### FcεRI-Induced Cytokine Production Is Defective in miR-155 KO BMMCs

**Figure 5:**
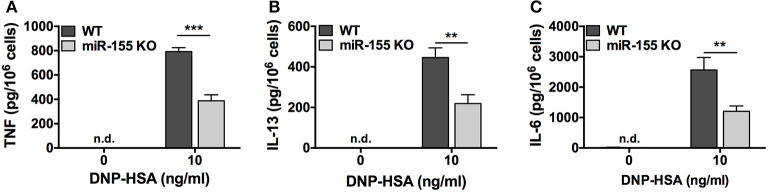
FcεRI-induced cytokine production is impaired in miR-155 KO BMMCs. Wild-type and miR-155 KO BMMCs were sensitized with anti-DNP-IgE and challenged with multivalent antigen DNP-HSA for 24 h. Secreted TNF **(A)**, IL-6 **(B)**, and IL-13 **(C)** in supernatant were measured by commercial ELISA. The bar graphs represent mean ± SEM of values obtained from independent experiments with three different BMMC cultures from different mice. Cytokines in untreated samples were generally below the level of detection. Lowest level of detection for TNF, IL-6, and IL-13, respectively, were 31.2, 15.6, and 62.5 pg/ml. n.d., not detected. Significance was determined with Student's *t*-test. **p < 0.01; ***p < 0.001.

We determined the effect of miR-155 deficiency on FcεRI-induced production of the proinflammatory cytokines TNF and IL-6, and the mucus-promoting cytokine IL-13, which plays a prominent role in asthma. BMMCs from wild-type and miR-155 KO mice were sensitized with anti-DNP IgE and challenged with DNP-HSA (10 ng/ml) for 24 h, and secreted cytokines in the cell-free supernatants was determined. As shown, TNF, IL-6, and IL-13 were detected at significantly lower levels in supernatants from miR-155-deficient BMMCs compared to WT cells (Figure 5). miR-155 KO BMMCs produced at least 50% less TNF, IL-6, and IL-13 compared to wild-type cells indicating that FcεRI-induced cytokine production was inhibited in the absence of miR-155.

**Figure 6:**
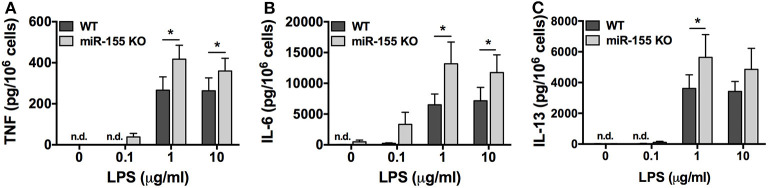
TLR4-induced cytokine production is enhanced in miR-155 KO BMMCs. Wild-type and miR-155 KO BMMCs were treated with 0.1, 1, or 10 μg/ml of LPS for 24 h and secreted TNF **(A)**, IL-6 **(B)**, and IL-13 **(C)** were measured by commercial ELISA. The bar graphs represent mean ± SEM of values obtained from independent experiments with three different BMMC cultures from different mice. Cytokines in untreated samples were generally below the level of detection. Lowest level of detection for TNF, IL-6, and IL-13, respectively, were 31.2, 15.6, and 62.5 pg/ml. n.d., not detected. Significance was determined with Student's *t*-test. *p < 0.05.

To determine if the observed inhibitory effect in the absence of miR-155 was specific to the FcεRI pathway or if the phenomenon was common to other signaling pathways, we compared the production of TNF, IL-6, and IL-13 from WT and miR-155 KO BMMCs after stimulation with LPS, which activates Toll Like Receptor 4 (TLR4). In dose-dependent studies, BMMCs were treated with LPS (0.1–10 μg/ml) for 24 h and secreted cytokines were measured by ELISA. In stark contrast to the inhibitory effect of miR-155 deficiency observed following FcεRI crosslinking, TLR4 activation with LPS resulted in significantly increased production of TNF, IL-6, and IL-13 from miR-155 KO BMMCs compared to their WT counterparts (Figure 6).

Together, these data reveal that miR-155 is a positive regulator of FcεRI-induced cytokine production but negative regulator of TLR4-induced cytokine synthesis in mast cells. These findings suggest the possibility that the target(s) of miR-155 might have different roles in TLR4 and FcεRI pathways. Moreover, the data suggest that the target of miR-155 in the FcεRI pathway is an inhibitor of cytokine production.

### FcεRI-Induced Phosphorylation of Akt Is Attenuated in miR-155 KO BMMCs

**Figure 7:**
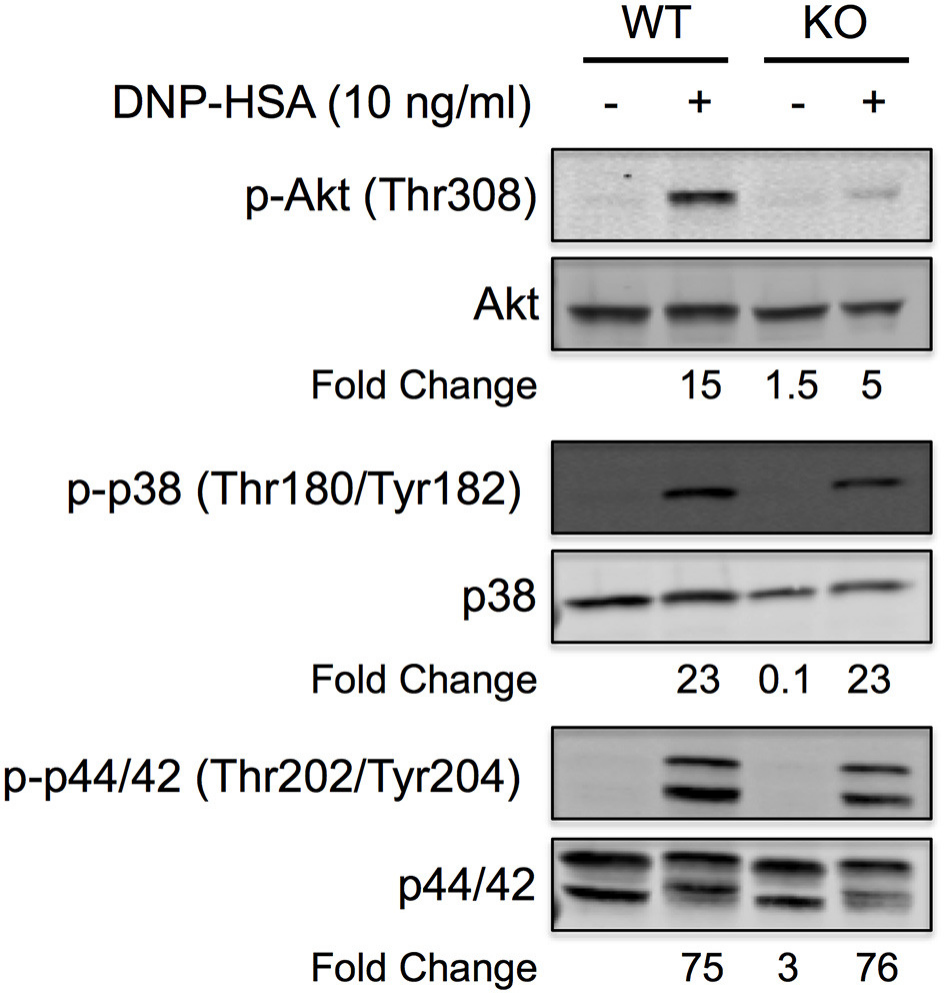
FcεRI-induced Akt phosphorylation is specifically inhibited in miR-155 KO BMMCs. Representative Quantitative Western blot showing phosphorylation status of Akt, p38, and p44/42 in wild-type and miR-155 KO BMMCs following FcεRI crosslinking with multivalent antigen. IgE-sensitized BMMCs were activated for 5 min with NP-BSA (10 ng/ml) and whole cell lysates were separated by reducing SDS-PAGE. Total and phosphorylated proteins were analyzed on the same blot by 2-color (680 and 800 nm) multiplexing on a near-infrared detection system. Blot is representative of at least three independent experiments with different BMMC cultures from different mice. Fold change in phosphorylation was calculated relative to non-activated wild-type BMMCs.

Akt is known to play a major role in FcεRI-induced cytokine production from mast cells, and BMMCs that lack Akt are defective in cytokine production (35). Therefore, to determine if Akt was involved in the defective cytokine production observed in Figure 6, we compared Akt expression and activation in WT and miR-155 KO BMMCs following FcεRI crosslinking. As shown, Akt protein was detected at similar levels in WT and miR-155 KO BMMCs indicating that Akt is not a direct target of miR-155. However, FcεRI-induced phosphorylation was severely attenuated in miR-155 BMMCs compared to WT BMMCs (Figure 7). As indicated in the representative figure, Akt phosphorylation was 3-fold lower in miR-155 KO BMMCs compared to WT BMMCs following FcεRI crosslinking. In contrast, phosphorylation of p38 and p42/44 (ERK1/2), which are known to play a role in FcεRI-induced activation of transcription factors and production of mast cell mediators (36), was unaffected by the absence of miR-155. Akt is known to be downstream in the phosphoinositide 3-kinase (PI3K) pathway and a direct substrate of phosphoinositide-dependent protein kinase 1 (PDK1) (36). However, we did not detect any difference in PI3K subunits p85 or p101, or PDK1 in miR-155 KO BMMCs (not shown) indicating that the defect in Akt phosphorylation in miR-155 KO BMMCs was not due to a deficiency in either of these kinases.

Taken together, these findings demonstrate that miR-155 positively regulates FcεRI-induced Akt activation. The data indicate that miR-155 does not directly inhibit Akt protein expression but suggest that miR-155 targets a phosphatase that normally inhibits Akt phosphorylation. In the absence of miR-155 the increased expression of the putative phosphatase leads to diminished Akt phosphorylation (as shown in Figure 7), which results in reduced cytokine production (as shown in Figure 5). The identity of the putative phosphatase remains to be determined.

## Discussion

Using miRNA seq analysis, we identified 10 miRNAs that were significantly (*p* < 0.01) upregulated and 11 that were significantly downregulated in human SMCs following FcεRI crosslinking. Similar analysis has been performed in mouse BMMCs (37–39) and some miRNAs have been reported to be involved in mast cell activation and allergic disease (20, 21). However, to our knowledge this is the first reported miRNA profile analysis in human SMCs. This study focused on miR-155, which was among the miRNA that were upregulated, because it had been reported that miR-155 expression in mouse BMMCs was stable and unaffected by FcεRI signaling (28). However, using quantitative RT-PCR, we provide clear evidence demonstrating that miR-155 expression increased following FcεRI crosslinking in both human and mouse mast cells. Thus, our study provides direct evidence that FcεRI signaling enhances the expression of miR-155 in mast cells thereby supporting the notion that miR-155 is involved in allergic disease where mast cells play a major role.

**Figure 8:**
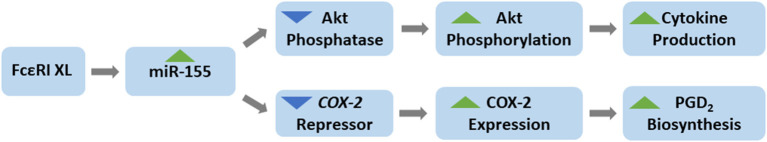
Schematic of the role of miR-155 in mast cells. FcεRI crosslinking (XL) induces miR-155 expression in mast cells. miR-155 acts to inhibit the expression of an Akt phosphatase and a *COX-2* repressor, thereby, facilitating Akt phosphorylation (activation) and *COX-2* expression which normally occur following FcεRI XL. Increased Akt phosphorylation (activation) and *COX-2* expression result in the production of cytokines and prostaglandins, respectively. Thus, miR-155 is a positive regulator of FcεRI-induced cytokine production and prostaglandin biosynthesis. miR-155 has no effect on degranulation or leukotriene production.

The major finding of the current study is that miR-155 specifically targets the FcεRI signaling pathways leading to prostaglandin biosynthesis and cytokine production but not the leukotriene or degranulation pathways (Figure 8). We found that FcεRI-induced *COX-2* expression and TNF, IL-6, and IL-13 production were significantly inhibited in miR-155 KO BMMCs compared with WT BMMCs whereas LTC4 biosynthesis and degranulation were unaffected by the absence of miR-155. Since miRNAs are negative regulators of gene expression, these data indicate that miR-155 acts indirectly by targeting one or more inhibitor(s) rather than a positive mediator of these pathways to positively regulate PGD_2_ biosynthesis and cytokine production.

Our data demonstrate a positive correlation between *COX-2* and miR-155 expression in mast cells following FcεRI crosslinking. A similar positive correlation in expression of miR-155 and *COX-2* has been reported in cancers, human airway smooth muscle, and mouse macrophages (40), and the question of whether and how miR-155 regulates *COX-2* expression and the complexity in miRNA regulation of *COX-2* gene expression has been documented (40, 41). Our finding that *COX-2* expression is inhibited in the absence of miR-155 when FcεRI signaling is fully intact demonstrates that miR-155 acts to enhance *COX-2* expression in mast cells. Moreover, because of the nature of miRNAs, the data suggest that miR-155 targets an inhibitor or repressor of *COX-2* expression such that the level or amount of repressor is diminished when miR-155 is present; thus, allowing *COX-2* expression to occur unimpeded. Conversely, when miR-155 is absent, as in miR-155 KO BMMCs, expression of the repressor is expected to be higher than normal and *COX-2* expression diminished as demonstrated in this study.

Similarly, we found that FcεRI-induced cytokine production was significantly inhibited in the absence of miR-155. Correspondingly, FcεRI-induced phosphorylation of Akt, but not protein levels, was significantly diminished in miR-155 KO BMMC compared to WT BMMCs. In the same vein as mentioned above, the data demonstrate that miR-155 is a positive regulator of cytokine production and suggests that miRNA acts indirectly by targeting an unknown phosphatase that normally inhibits Akt phosphorylation in the FcεRI pathway. We propose that miR-155 negatively regulates expression of the putative phosphatase thereby lowering the amount of phosphatase available to dephosphorylate Akt; thus, allowing phosphorylation of Akt to occur with minimal impediment. On the other hand, when miR-155 is absent, as in miR-155 KO BMMCs, the putative phosphatase is expected to be expressed at abnormally high levels resulting in enhanced dephosphorylation activity and diminished Akt phosphorylation as the data presented here demonstrate. A critical role for Akt in FcεRI-induced cytokine production in mast cells is well-established (35). Therefore, our finding that Akt phosphorylation is significantly inhibited in miR-155 KO BMMCs aligns with the observation that cytokine production is also significantly diminished in the absence of miR-155.

It is worth noting that the findings of our study contrast with those of Biethahn et al. (28), which showed increased production of cytokines and Akt phosphorylation in miR-155 KO compared to WT BMMCs. The reason for this discrepancy is unknown. Throughout the course of our study, we confirmed the genotype of the BMMCs as demonstrated in Figure 3 to rule out any possible mix-up. Noteworthy, we found that stimulation with LPS resulted in increased cytokine production from miR-155 KO compared with WT BMMCs. However, any potential issue with contamination is purely speculative, but, nevertheless, worth mentioning considering our differential results with FcεRI and LPS stimulation. It is also worth noting that, in contrast to our findings, an earlier study found decreased LPS-induced cytokine production from miR-155 KO BMMCs compared to WT cells (42). In addition, a separate study from the same lab found no significant difference in IgE-dependent cytokine production between miR-155 KO and WT BMMCs and also indicated that there was no difference in the *in vivo* IgE-dependent anaphylaxis response (29). The reason for these discrepancies is not known. However, it is worth mentioning that the BMMCs used in those studies were cultured in complete RPMI media supplemented with IL-3-containing medium from WEHI-3 cells and SCF-containing media from BHK-MKL cells whereas our studies used only complete RPMI media supplemented with purified recombinant IL-3 and SCF. Another possibility, particularly with regard to the *in vivo* anaphylactic response, is that there are differences in microbiome, which includes not only bacteria but also viruses, within the animals housed in the different animal facilities. Although speculative, this possibility is intriguing because the microbiome has been shown to have the capacity to influence the development of allergic disease (43). It remains to be determined if differences in culture conditions and/or the microbiome is the reason for the differential observations. Our finding that miR-155 expression is increased following FcεRI crosslinking aligns with the observation that miR-155 is elevated in tissues affected by allergic disease which suggests that miR-155 potentiates the pathogenesis of the disease. Given this observation and the nature of miRNAs as negative regulators of gene transcription, it is reasonable to assume that a deficiency in miR-155 would result in diminished rather than enhanced production of allergic mediators, which is what our study indicates.

Our study demonstrates that miR-155 expression facilitates the production of mediators of allergic inflammation. Interestingly, infections with human rhinoviruses, which are known to exacerbate asthma, have also been shown to be associated with increased miR-155 in the airways (44, 45). It is thought that viruses themselves cause the release of extracellular vesicles carrying miR-155 (44, 46). It has been further speculated that the increase in miR-155 in the airways following rhinovirus infection could be a mechanism by which these viruses promote asthma pathogenesis. Our findings that miR-155 promotes the production of prostaglandins and cytokines involved in asthma pathogenesis is one potential mechanism by which rhinoviruses might act to exacerbate asthma and lend support to the notion that the additional presence of miR-155 following viral infection could contribute to asthma pathology. It is also worth noting that miR-155 expression in mast cells has also been shown be increased by IL-10 (47). IL-10-induced miR-155 was shown to enhance the production of cytokines and proteases that could contribute to the exacerbation or development of airways disease (29). These studies and ours begin to reveal a mechanism in which miR-155 acts to promote airways disease by potentiating the release of mediators of allergic inflammation from mast cells.

Overall, our study provides evidence that miR-155 is a positive regulator of the prostaglandin and cytokine pathway in mast cells and that it acts by targeting inhibitors of COX-2 expression and Akt phosphorylation. Additional studies are necessary and underway to identify and characterize the putative inhibitors. These studies include, for example, Ingenuity Pathway Analysis and other *in silico* tools to identify potential targets that map to miR-155, use of miR-155 mimics and inhibitors to modulate miR-155 expression, and biochemical studies to determine if expression of the potential targets correlate with miR-155 expression. These studies will identify the targets of miR-155 and provide additional mechanistic data to further explain how miR-155 functions in mast cells and potential role in allergic disease.

## Data Availability Statement

The complete datasets presented in this study can be found in the Supplementary Material.

## Ethics Statement

The animal study was reviewed and approved by Institutional Animal Care and Use Committee (IACUC) of the University of South Carolina.

## Author Contributions

ZM performed the experiments and assisted with data analysis. CM assisted with the experiments. JK performed the flow cytometry analysis. SD assisted with writing and reviewing the manuscript. GG was principal investigator of the project, directed the project, analyzed the data, and wrote the manuscript. All authors contributed to the article and approved the submitted version.

## Funding

This study was funded by National Institutes of Health grant 1P20GM103641 (Project 4) (GG), a fellowship from the Higher Committee for Education Development (HCED) and Ministry of Higher Education and Scientific Research (MOHSR) in Iraq (ZM), and American Association of Immunologists (AAI) Careers in Immunology Fellowship (GG and CM).

## Conflict of Interest

The authors declare that the research was conducted in the absence of any commercial or financial relationships that could be construed as a potential conflict of interest.

## Publisher's Note

All claims expressed in this article are solely those of the authors and do not necessarily represent those of their affiliated organizations, or those of the publisher, the editors and the reviewers. Any product that may be evaluated in this article, or claim that may be made by its manufacturer, is not guaranteed or endorsed by the publisher.
